# Psychotropic and anti-epileptic drug use, before and after surgery, among patients with low-grade glioma: a nationwide matched cohort study

**DOI:** 10.1186/s12885-021-07939-w

**Published:** 2021-03-08

**Authors:** Isabelle Rydén, Erik Thurin, Louise Carstam, Anja Smits, Sasha Gulati, Roger Henriksson, Øyvind Salvesen, Asgeir Store Jakola

**Affiliations:** 1 Department of Clinical Neuroscience, Institute of Neuroscience and Physiology, University of Gothenburg, Sahlgrenska Academy, Blå stråket 7, 3 tr, 413 45 Gothenburg, Sweden; 2 Department of Neurosurgery, Sahlgrenska University Hospital, Gothenburg, Sweden; 3 Department of Neurosurgery, St.Olavs University Hospital HF, Trondheim, Norway; 4 Department of Radiation Sciences and Oncology, University of Umea, Umeå, Sweden; 5 Department of Public Health and Nursing, Norwegian University of Science and Technology, Trondheim, Norway

**Keywords:** Glioma; low-grade glioma, Antidepressive agents, Anti-anxiety agents, Anticonvulsants

## Abstract

**Background:**

Low-grade glioma (LGG) is a relatively rare type of brain tumour. The use of antidepressant, sedative and anti-epileptic drugs can reflect the burden of the disease. While epilepsy is well-described in patients with LGG, less is known about depression and anxiety.

**Methods:**

We used nationwide registers to study the use (dispense) of antidepressants, sedatives, and anti-epileptic drugs (AEDs) before and after histopathological LGG diagnosis (WHO grade II). A total of 485 adult patients with a first-time diagnosis and a matched control cohort (*n* = 2412) were included. Patterns of use were analysed from one year prior to until one year following index date (date of surgery). Logistic regression analysis identified predictors for postoperative use.

**Results:**

At one year before index date, patients were dispensed AEDs 4 times more than controls, while antidepressants and sedatives were similar. Sedatives and AED peaked shortly after index date at 25 and 69%, respectively. AEDs then stabilized while sedatives decreased rapidly. For antidepressants, a delayed increase was seen after index date, stabilizing at 12%. At one year after index date, the use of antidepressants, sedatives, and AEDs among patients was 2, 3, and 26 times higher, respectively, compared to controls. Predictor for use of AEDs and sedatives at one year following index was previous use and/or a related diagnosis. Female sex and later index year were additional predictors for antidepressants.

**Conclusions:**

Use of antidepressants, sedatives and AEDs is elevated following diagnosis of LGG. Antidepressants were more commonly dispensed to female patients and in recent years.

**Supplementary Information:**

The online version contains supplementary material available at 10.1186/s12885-021-07939-w.

## Background

Diffuse low-grade gliomas (LGG) (WHO grade II) are relatively rare types of primary brain tumours, typically affecting younger adults. The majority of patients present with epileptic seizures [1, 2] and receive anti-epileptic drugs (AEDs) [3, 4]. In spite of optimal AED treatment, seizure control can be difficult to accomplish [2, 4, 5]. In the general epilepsy population, uncontrolled seizures are associated with a higher risk for developing depression and anxiety [6]. Furthermore, depression and anxiety can be side effects of the AED treatment [7].

A cancer diagnosis increases the risk for developing depression [8]. If depression is left untreated the risk of recurrence increases [9]. The presence of depression and anxiety significantly reduces patients’ quality of life and can even result in suicide [10]. As recently demonstrated, patients with glioblastoma were four times more likely to commit suicide within the first year after diagnosis, compared to patients with less malignant brain tumour types, including LGG, who had a doubled risk compared to the general population [11]. Studies on the prevalence of depression in patients with brain tumours have reported large variations (range 0–93% [12, 13]). Hence, reviews indicate that approximately 15–20% of patients with brain tumours develop depression [12, 13]. Only a few smaller studies have studied depression and anxiety in patients with low-grade tumours [14, 15]. Much of the data on depression and anxiety in patients with LGG come from studies including patients with brain tumours of different types and grades, applying various methodologies, thus making it difficult to draw conclusions for the group of LGG in specific [16–20]. An intriguing way to obtain further data is to study dispense of sedatives (anxiolytics, hypnotics and sedatives) and antidepressants, which reflects the use of these drugs in the LGG population and can serve as indicators of depression and anxiety disorders. By this approach we may also be able to address potential discrepancies between expected symptom burden and treatment provided [17].

The aim of our study was to investigate temporal patterns of the use of psychotropic drugs and also AEDs in patients with LGG around time for diagnosis compared to a matched control group. In addition, we explored predictors for drug use in order to investigate factors associated with changes in use.

## Methods

The unique personal identification numbers of Swedish citizens enabled Statistics Sweden (SCB) to link data from several national Swedish registries, and to create a matched control group. The registries are described below.

Patients were identified via the Swedish brain tumour registry (SBTR). Data on patients ≥18 years with a histopathologically verified first-time diagnosis of LGG between January 1, 2005 until December 31, 2015, were derived. LGG was defined as grade II astrocytoma, oligoastrocytoma or oligodendroglioma according to the 2007 WHO classification of brain tumours [21].

### The Swedish brain tumour registry

The SBTR is the National Quality Registry for Brain Tumours. It is regionally based covering adult patients diagnosed with brain tumours. SBTR carries detailed information on patient- and tumour-related characteristics. For further details of the registry, see Asklund et al. [22]. The level of coverage has varied between centres over time. Since we aimed for population-based data without significant selection bias, we set a minimum registration rate of 80% to be included. This rate was defined as the percentage of diagnoses (evaluated per year) corresponding to diagnoses reported to the compulsory National Cancer Registry. Consequently, data from 2005 to 2011 was excluded for one centre and from 2005 to 2008 from another centre. The estimated loss of patients, based on the report of patients during the surrounding years, was found to be approximately 165 for the excluded period. The four remaining centres were included for the entire study period. Data from SBTR was accessed October 21, 2016.

### Statistics Sweden (SCB)

SCB is a government agency responsible for coordinating the system for official statistics in Sweden. From the SCB, we extracted data on education and income. Educational level was graded according to the Swedish nomenclature for education [23] and divided into two groups: basic to high-school (SUN2000 grade 1–4) and higher education (SUN2000 grade 5–7). A matched control cohort of five unique individuals per case was obtained, all matched by year of birth, sex, educational level, and municipality of residence. The cohort of matched controls was incomplete due to lack of individuals with matching criteria, resulting in 13 missing control cases. This generated a control population of 2412 individuals. Data from Statistics Sweden was accessed June 26, 2017.

### The National Board of Health and Welfare (NBHW)

NBHW is a government agency responsible for several national registries. We used data from two of these registries: the *National Patient Registry* (NPR) and the *National Prescription Registry* (NPrR). The registries under the NBHW were both accessed January 8, 2018.

Reporting to the NPR has been mandatory since 2001. The NPR contains information about all contact with specialist health care with diagnoses coded according to the 10th revision of the International Classification of Diseases (ICD-10). We used the ICD-10 codes to classify comorbidities according to the Elixhauser comorbidity index [24], with the exclusion of conditions associated with LGG, as defined in Table 1.

**Table 1:** Definition of variables

Variable	Definition
**Index date**	Date of surgery
**Index year**	Year of surgery
**Use of anti-epileptics (AED)**	Purchase of ATC class N03A (anti-epileptics), except those mainly used for pain: N03AX12 (Gabapentin) and N03AX16 (Pregabalin), within the last 90 days.
**History of seizure/epilepsy**	Purchase of ATC class N03A (anti-epileptics), except N03AX12 and N03AX16 and/or ICD-10: G40 (epilepsy) and/or registered “seizure” as symptom in SBTR. All within one year prior to index date.
**Use of antidepressants**	Purchase of ATC: N06A (antidepressants) within the last 90 days.
**History of depression**	ATC: N06A and/or ICD-10: F20.4 (post-schizophrenic depression), F31.3-F31.5 (bipolar affective disorder), F32 (depressive episode), F33.0-F33.3 (recurrent depressive disorder), F34 (persistent mood disorders), F41.2 (mixed anxiety and depressive disorder), F43.2 (adjustment disorders). All within one year prior to index date.
**Use of sedatives**	Purchase of ATC: N05B (anxiolytics) and N05C (hypnotics and sedatives) within the last 30 days.
**History of anxiety and/or sleeping difficulties**	ATC: N05B and/or N05C (except N05CD08) and/or ICD-10: G47 (sleeping disorders), F40 (phobias), F41.0 (anxiety disorders), F41.1, F41.3, F41.8, F41.9, F42.0 (obsessive disorders), F42.1, F42.2, F42.8, F42.9. All within one year prior to index date.
**Elixhauser comorbidity index**	According to index. The conditions removed from the index due to possible association with diagnosis of LGG were: G40, G41 (Status epilepticus), R56 (Convulsions), R47 (Dysphasia/aphasia), C70–72 (Malignant tumour in central nervous system). Cases and controls were provided with a score from 0 to 30 based upon comorbid categories present or not. We report categories as 0, 1, 2, ≥3. The ICD-10 data used to classify comorbidity was taken from the NPR during the year prior to index date.

The underreporting to the NPR has been estimated to be 1% [25]. We received data on inpatient and outpatient visits, including diagnostic and procedural codes in the period January 1, 2003 - December 31, 2016.

The NPrP covers all prescribed drugs delivered by a pharmacy. Registration is mandatory. We received information on type of drug according to the Anatomical Therapeutic Chemical (ATC) classification system and date of dispensing between July 1, 2005 until December 31, 201**7**. We retrieved information on all anti-epileptic (N03A), sedative (anxiolytics, hypnotics and sedatives: N05B and N05C) and antidepressant drugs (N06A). We excluded drugs and diagnoses commonly used for other conditions, when creating variables to the regression models. Use was defined as having purchased at least one of the specified drugs within the last 90 days for AEDs and antidepressants, and 30 days for sedatives (prescriptions of sedatives are usually for shorter periods). Definitions of variables are presented in Table 1.

Since the NPrR was established July 1, 2005, all patients with index dates before October 1, 2006 were excluded (*n* = 58). Patients that had moved abroad (*n* = 4), were not able to match with controls and thus excluded. Patients in the remaining group (*n* = 485) were available for analyses at index date. All patients alive at 1 year postoperative were analysed in the regression models (*n* = 435). Patient selection is presented in Supplement Fig. 1.

### Statistics

**Fig. 1:**
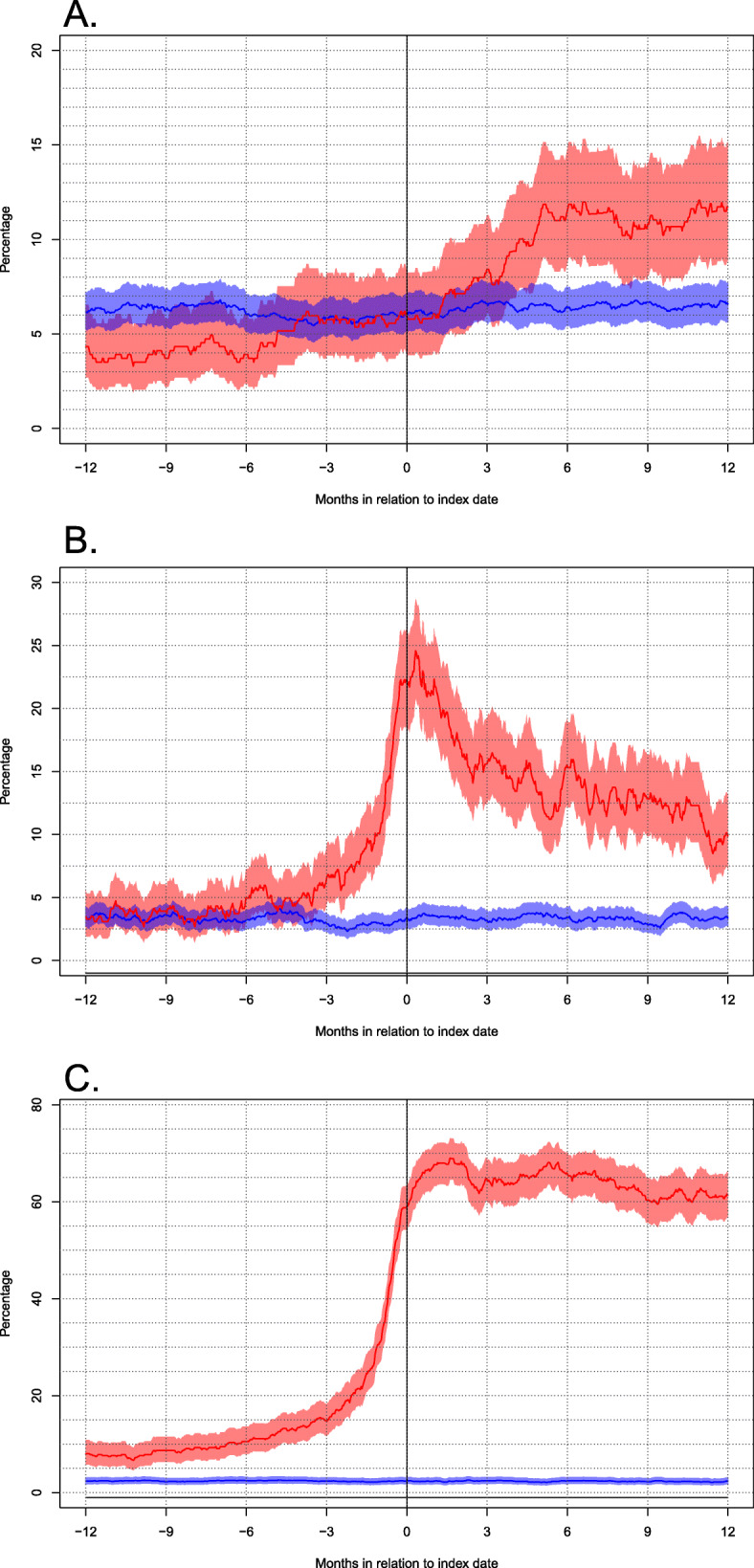
**a**, **b**, **c** Graphs demonstrating the proportion of patients with LGG (red) (95% CI) versus controls (blue) with a use of antidepressants (**a**), sedatives (anxiolytics and hypnotics) (**b**) and anti-epileptics (**c**) in relation to time from one year prior to index date through one year following index date

Data from the registries was imported into a mySQL (Oracle) database. Drug dispense were individually analysed for each patient (date and ATC-code) and combined with clinical data using Python. R statistical software version 3.1 was used for statistical analyses. For each day from 1 year prior to, until 1 year following index date, the proportion of patients classified as users of the drugs included in the different prescription groups was calculated and displayed as graphs with patients and controls, as well as confidence intervals (Fig. 1a, b, c).

Continuous variables were summarized using the median, first and third quartiles and compared between cases and controls using the Mann-Whitney U test. Categorical variables were summarized using counts and proportions and compared between cases and controls using the Fisher’s exact test. Univariable and multivariable logistic regression analyses were done using SPSS 25.0. Covariates were chosen based upon presumed relevance. All tests were two-sided and statistical significance level was set to a *p*-value < 0.05.

## Results

### Demographic data

Based on the selection presented above, 485 patients were included at index date. The mean age was 46 years and 44% were females. The majority could perform at least light work (82%). Of patients, 72% had experienced an epileptic seizure or had been dispensed AEDs during the previous year. Corresponding numbers for antidepressants and/or diagnosis of depression were 13, and 26% for anxiety and/or sleeping difficulties. Further baseline and treatment characteristics for patients are presented in Table 2.

**Table 2:** Baseline and treatment characteristics for patients with LGG (*n* = 485)

Variable	All patients
Age, mean (SD)	46 (14.7)
Female, n (%)	215 (44)
Days from imaging diagnosis to surgery, median (Q1-Q3)	37 (21–90)
WHO performance status, n (%)	
0. Fully active	263 (55.6)
1. Light work possible	124 (26.2)
2. Cares for self but cannot work	65 (13.7)
3. Limited self care	16 (3.3)
4. Disabled, confined to bed/chair	5 (1.1)
Missing, n	12
Tumour laterality, n (%)	
Left	250 (52.2)
Right	209 (43.6)
Bilateral	20 (4.2)
Missing, n	6
Tumour size	
< 4 cm	177 (40.0)
4–6 cm	174 (39.4)
> 6 cm	91 (20.6)
Missing, n	43
History of seizure/epilepsy, n (%)	351 (72.4)
Missing, n	0
History of depression, n (%)	61 (12.6)
Missing, n	0
History of anxiety and/or sleeping difficulties	126 (26.0)
Missing, n	0
Surgical procedure, n (%)	
Biopsy	131 (27.3)
Resection	349 (72.7)
Missing, n	5
New deficit after surgery, n (%)	84 (18.9)
Missing, n	40
Reoperation due to complication, n (%)	27 (6.1)
Missing, n	41

Supplement Table 1 shows a comparison of patients and controls. As seen, patients had a significantly higher number of comorbidities (*p* < 0.001), however, 92% of patients had none or only one comorbidity, compared to 94% in the control group. Level of education and disposable income (used as matching criteria) did not differ between the groups.

### Antidepressants

Figure 1a shows a graph comparing proportions of users of antidepressants (95% CI) between patients and controls during the study period. No differences between groups were seen before index date. The peak in use among patients was seen at 5 months after index date (12%) with a doubled use compared to controls. Use among patients remained stable (≈10–12%) until the end of follow up. Controls remained around 6% during the entire study period.

Among patients using antidepressants at index date, 48.3% were still using the drug at 1 year following. Interestingly, new users represented a large proportion of patients classified as “users at one year after index”; 37 out of 51 patients (72.5%). Detailed overview of changes is presented in Supplement Table 2.

Predictors for use of antidepressants among patients at 1 year after index were explored in regression models (Table 3). In the multivariable model, history of depression (*p* < 0.001), female sex (*p* = 0.001) and later index year (*p* = 0.002) were significant predictors at 1 year.

**Table 3:** Logistic regression model for use of antidepressants, sedatives and anti-epileptics, respectively, at one year following index date, for patients with LGG

Covariate	Univariable		Multivariable	
	OR (95 % CI)	***p***-value	OR (95 % CI)	***p***-value
**ANTIDEPRESSANTS**				
Index year (per year)	1.22 (1.07-1.39)	0.002**	1.31 (1.11-1.55)	0.002**
Female (vs. male)	2.99 (1.60-5.60)	0.001**	3.41 (1.71-6.80)	0.001**
Age (per year)	1.02 (1.00-1.05)	0.027*	1.02 (1.00-1.05)	0.11
Income (per 100.000 SEK)	0.96 (0.76-1.20)	0.69	1.07 (0.83-1.39)	0.60
Higher education (vs. lower education)	0.94 (0.78-1.15)	0.56	0.97 (0.78-1.21)	0.78
History of depression	7.61 (3.88-14.94)	<0.001***	6.12 (2.96-12.63)	<0.001***
Use of antidepressants preoperatively	14.15 (5.88-34.08),	<0.001***		
Elixhauser comorbidity index (0, 1, 2, >3)	1.33 (0.96-1.84)	0.09	1.09 (0.75-1.59)	0.65
Functional level (per WHO performance status category)	0.98 (0.92-1.03)	0.38	0.96 (0.88-1.06)	0.42
Tumour size (6)	1.08 (0.96-1-21)	0.22	0.92 (0.79-1.07)	0.30
**SEDATIVES**				
Index year (per year)	1.13 (0.99-1.29)	0.07	1.12 (0.97-1.30)	0.14
Female (vs. male)	1.23 (0.66-2.30)	0.51	1.13 (0.58-2.19)	0.73
Age (per year)	1.02 (1.00-1.04)	0.09	1.02 (0.99-1.04)	0.24
Income (per 100.000 SEK)	0.87 (0.67-1.13)	0.30	0.90 (0.62-1.22)	0.49
Higher education (vs. lower education)	0.81 (0.66-1.00)	0.05	0.90 (0.71-1.13)	0.35
History of anxiety and/or sleeping difficulties	2.58 (1.36-4.90)	0.004**	2.14 (1.09-4.20)	0.027*
use of sedatives preoperatively	3.05 (1.60-5.82),	<0.001***		
Elixhauser comorbidity index (0, 1, 2, >3)	1.37 (0.98-1.92)	0.07	1.17 (0.80-1.70)	0.41
Functional level (per WHO performance status category)	0.97 (0.91-1.04)	0.42	0.97 (0.89-1.05)	0.43
Tumour size (6)	1.09 (0.97-1.23)	0.17	1.00 (0.87-1.16)	0.98
**ANTI-EPILEPTICS**				
Index year (per year)	1.13 (0.99-1.29)	0.07	1.12 (0.97-1.30)	0.14
Female (vs. male)	1.23 (0.66-2.30)	0.51	1.13 (0.58-2.19)	0.73
Age (per year)	1.02 (1.00-1.04)	0.09	1.02 (0.99-1.04)	0.24
Income (per 100.000 SEK)	0.87 (0.67-1.13)	0.30	0.90 (0.62-1.22)	0.49
Higher education (vs. lower education)	0.81 (0.66-1.00)	0.05	0.90 (0.71-1.13)	0.35
History of anxiety and/or sleeping difficulties	2.58 (1.36-4.90)	0.004**	2.14 (1.09-4.20)	0.027*
use of AED preoperatively	9.49 (6.07 – 14.85),	<0.001***		
Elixhauser comorbidity index (0, 1, 2, >3)	1.37 (0.98-1.92)	0.07	1.17 (0.80-1.70)	0.41
Functional level (per WHO performance status category)	0.97 (0.91-1.04)	0.42	0.97 (0.89-1.05)	0.43
Tumour size (6)	1.09 (0.97-1.23)	0.17	1.00 (0.87-1.16)	0.98

*p <0.05, **p < 0.01, ***p < 0.001

We performed post-hoc explanatory analyses on the impact of sex and index date, separately, in relation to use of antidepressants. At index date, 8.4% of female and 4.1% of male patients were using antidepressants. Corresponding rates in controls were similar: 8.3% in females and 4.3% in males. At 1 year following index date, the use of antidepressants had mildly increased in male patients (6.7%), whereas female patients had doubled their use (17.8%). Corresponding rates in controls were: 8.7% for females and 5.0% in males at 1 year.

A comparison of proportions of users of antidepressants in relation to index year was presented for patients and controls (Supplement Table 3). There was a marked increase in use for the last years of the inclusion period, where 20.8% of patients diagnosed in 2015 and 11.4% of controls used antidepressants 1 year after index date, (Supplement Table 3).

### Sedatives

Figure 1b presents a comparison of proportions of users of sedatives in patients and controls. The use of sedatives did not differ between patients and controls at 1 year before index date. An exponential increase was seen among patients close to index date, peaking at 24.6%, followed by a rapid decrease. At 1 year after index date, the use was 9.8% among patients (three times higher compared to controls). The control group showed a stable use around 3% during the entire study period.

Supplement Table 4 shows the change in users of sedatives over time. Of patients using at index date, 17.6% were still using 1 year after index date.

Regression models for prediction of use of sedatives at 1 year following index are presented in Table 3. Both in the univariable (*p* < 0.001) and multivariable (*p* = 0.027) regression model, we identified “history of anxiety and/or sleeping difficulties” as the only predictive variable.

### Anti-epileptic drugs

As seen in Fig. 1c, patients had a significantly higher use of AEDs 1 year prior to index date (8% compared to 2%). A further increase was seen among patients approximately half a year before index date, with an exponential increase during the last months before index date. At index date, 59% of the patients were using AEDs. The peak for AEDs was seen at 1.5 months after index date (69%), followed by a fairly stable use up to 1 year after index date (61%). The use in the control group remained at around a level of 2% during the entire study period.

The majority of patients using AEDs at index date also used it at 1 year following (75.4%) (Supplement Table 5). A switch of groups (from use to no use and vice versa) was seen for 50 patients with previous use, and for 53 patients without previous use.

Predictors for use of AEDs among patients at 1 year after index date, were explored using regression models (Table 3). The “history of seizure/epilepsy” was the only significant factor of the predictors included.

### Sensitivity analyses

The definitions of “history of epilepsy”, “history of depression” and “history of anxiety/sleeping difficulties” were analysed in sensitivity analyses by separating diagnoses (NPR) and specified drugs (NPrP) and analysing them as separate variables in the regression models. These analyses revealed each variable to be individually significant; hence the use of different definitions did not alter the results. The related diagnoses and drugs are specified in Table 1.

## Discussion

### Antidepressants

The average use of antidepressants in Europe for 2010 was 7.2%, with Sweden being close to this average [26]. In our material, patients and controls had similar rates of antidepressant use, i.e. around 6% at index date. Controls remained stable, but the use increased for patients following index date, peaking at 12% at 5 months. This is almost identical to what has been reported in a previous study on antidepressants in patients with various types of low-grade brain tumours (12.2%) [14]. Our results indicate that pharmacological treatment for depression is typically initiated months following surgery.

Previously, the Glioma Outcomes Project found a discrepancy both between the use of antidepressants, and between rates made by patients and clinicians, where patient’s rates were higher [17]. These discordances were most pronounced directly after surgery, but still significant at 6 months postoperatively, suggesting a possible under-treatment of these patients [17]. The use of antidepressants in our material was elevated compared to controls, yet somewhat lower than what would be expected from previous studies [12].

Rates of depression have suggested to increase as the disease progresses, also in patients with LGG [14]. On the contrary, a smaller study on patients with mixed primary brain tumours showed a stable pattern, with 16% of patients classified as depressed preoperatively and 15% at 1 year postoperatively [27]. Thus, this study reported a higher rate of preoperative depression, but a relatively similar rate of postoperative depression, compared to our data on the use of antidepressants.

Previous studies have shown a history of depression [20, 28] and other psychiatric illness [20, 29] to be related to a higher risk for developing depression and anxiety in a postoperative phase. Our study confirmed these findings. Furthermore, female patients had an elevated use of antidepressants 1 year after index, both compared to male patients (2.7 times) and female controls (2.0 times). Overall, we found that depression [30] and the use of antidepressants [26] were more common among females compared to males, with approximately doubled incidence for women, and a doubled use of both antidepressants and anxiolytics [31]. A similar pattern has been reported for patients with brain tumours overall [29, 32–34] and studies specifically addressing patients with various types of low-grade tumours have confirmed this [14, 15]. Thus, our results are in line with previous studies and provide additional knowledge of an increased risk for use of antidepressants in the female population of LGG.

In our material, both patients and controls with later index dates had a higher use of antidepressants, compared to those with earlier index dates. This tendency of increased use in later years is a novel finding. We hypothesize that the discrepancy in patient reported symptoms and antidepressant use have decreased during the last years since the more recent numbers match the percentage reporting symptoms well. This may reflect an increased awareness of symptoms and/or changed attitudes towards pharmacological treatment of psychiatric symptoms during recent years, since the use also has increased in the general population over time [35].

### Sedatives

Symptoms of anxiety are more common among cancer patients compared to the general population [36]. Sedatives, including anxiolytics and hypnotics, are given to relieve symptoms of anxiety, but also for sleeping difficulties. The large drug group benzodiazepines can cause dependence and withdrawal symptoms and are therefore preferably prescribed for short term use. Naturally, this explains our findings of an increased use of sedatives in patients around index date and the relatively rapid decrease that was noticed afterwards. Still, it is not surprising that some patients need sedatives for longer periods of time. In our study, patients had an excess use at 1 year postoperative compared to controls, indicating higher levels of these symptoms among patients even after a year. This increased use might reflect the patient’s level of anxiety, but could possibly also be explained by other factors, such as a more liberal view in prescribing sedatives to patients with cancer.

“Previous use and/or diagnosis” was the only significant predictive variable in the multivariable analysis for use of sedatives. Few patients were previously naïve users at 1 year after index, indicating that a proportion of patients have a prolonged use and possibly also find it difficult to stop medication of these drugs. Interestingly, female sex was not a significant predictor.

### Anti-epileptic drugs

Patients in the present study had a higher rate of AED use compared to controls already at one year prior to index date (8% compared to 2%). The exponential increase in use of AEDs in our study corresponds well with epileptic seizures being the most common first symptom of LGG, and with the median wait time of 37 days from radiological diagnosis to surgery. The peak in use at 69% is in line with previous studies on patients with low-grade tumours before start of oncological treatment [14]. The proportion of users among patients remained stable until end of follow up, indicating no major changes in usage during this period. This likely indicates a lack of symptom control without use of AEDs, as well as a tradition of not tapering out AED in the setting of a chronic condition even when symptom control is achieved, for instance after gross total resection [3]. This assumption is strengthened by the fact that type of surgery did not predict long-term use. It is our clinical experience that both patients and clinicians are careful in reducing AEDs, especially during the first year to stay on the safe side avoiding epileptic seizures. Patients can also be eager on getting their driving license back (usually retracted for 1 year after the last seizure) and do not want to risk having the retraction prolonged. However, overtreatment of AEDs may lead to unnecessary exposure to well-known side effects such as cognitive deficits, fatigue, and psychiatric symptoms [37]. This has to be weighed against risk of recurrence of seizure and the abovementioned consequences in terms of use of machinery and driving restrictions. For some patients, this may also directly influence their ability to return to work**.** Due to this balancing act, the potential tapering of AEDs in some instances should be well suited for shared decision making, since priorities may largely vary between patients.

The patterns of use of AEDs indicates a potential need for a more individual evaluation, especially following gross total tumour resection, and in case of tumours with favourable molecular profiles where longer-term tumour control can be achieved.

### Study limitations

We have tried to identify specific drugs used to treat depression, anxiety/sleeping disorders, and epilepsy. However, symptoms of the psychiatric conditions are largely overlapping and these drugs can be prescribed for other conditions. To derive specific diagnoses from the use of these drugs is therefore a simplification. Although it may be somewhat artificial, the temporal trends correspond reasonably well with the expected use for the assumed indications and findings are supported by our sensitivity analysis (analysing diagnoses vs. medications). It should also be emphasized that dispensing is not necessarily equal to an actual use during the period described. Yet, purchasing a prescribed drug is a more specific measure than simply counting prescriptions. The assumption of active use is further strengthened by the fact that the same patient often repeatedly bought the same drugs (most prominent for antidepressants and AEDs).

As with all registry studies, data is limited in type and details of variables, such as molecular data, but registry data has the major benefit of high coverage and limiting bias from geographic and socioeconomic sources. It is also a great strength that it is possible to link various data from relevant registries (e.g. quality registry with prescriptions and diagnoses). Importantly, we were also able to evaluate potential excess use by comparing results to a large and well-matched control group. Also, in instance on dispensed prescriptions, we have a high temporal resolution. Because the registry is based on mandatory national registries, patients are not lost at follow up due to unwillingness to participate or discontinuing. This is of particular importance when studying drugs for depression and anxiety, where loss of interest or avoiding contact may be key symptoms.

Since we cannot study factors in isolation, it is difficult to make any conclusions on the effect of the diagnosis and the disease itself, or the effect caused by treatment or other unrecognized factors.

## Conclusions

We found that the use of antidepressants, sedatives and AEDs in patients with LGG was elevated at 1 year following surgery, compared to matched controls. Previous use, or a related diagnosis motivating treatment with these drugs, were the most prominent predictors for use at 1 year after index date, for all categories. Female sex and later index year were identified as additional predictors for antidepressants.

Our study stresses the importance of asking patients with LGG about mental health and to consider pharmacological treatment or referral to psychiatric or psychological help, since depression and anxiety can substantially impair the quality of life. A lower threshold for initiating antidepressant treatment and a smaller discrepancy between use of antidepressants and prevalence of depression was seen during the last years, possibly reflecting an increased attention, or willingness to treat depressive symptoms with antidepressants. Even though most users of sedatives had intended short-term use, there was an increased long-term use among patients with previous use. A more cautious use of sedatives “pro re nata” to achieve an improvement in prescription pattern may be advocated. The stable use of AEDs over time, even when gross total tumour resection is achieved, may indicate over treatment in some situations. The potential withdrawal of AEDs should be subject to shared decision making.

## Supplementary Information

**Additional file 1: Supplement Table 1.** Characteristics of patients and controls. **Supplement Table 2.** changes in use of antidepressants for patients and controls. **Supplement Table 3.** Proportion (%) of users of antidepressants at one year after index date in relation to index year. **Supplement Table 4.** changes in use of sedatives for patients and controls. **Supplement Table 5.** changes in use of AEDs for patients and controls.

**Additional file 2: Supplement Fig. 1**. Flow chart of patient selection.

## Data Availability

The data that support the findings of this study are available from the registry holders (The Swedish brain tumour registry, Statistics Sweden, and The National Board of Health and Welfare) but restrictions apply to the availability of these data, which were used under license for the current study, and so are not publicly available.

## Abbreviations

AED: Antiepileptic drugs
ICD-10: International Classification of Diseases
LGG: Low-grade glioma
NBHW: The National Board of Health and Welfare
NPR: National Patient Registry
NPrP: National Prescription Registry
SBTR: Swedish Brain Tumour Registry
SCB: Statistics Sweden
