# Correction: Psychotropic and anti-epileptic drug use, before and after surgery, among patients with low-grade glioma: a nationwide matched cohort study

**DOI:** 10.1186/s12885-022-09400-y

**Published:** 2022-03-31

**Authors:** Isabelle Rydén, Erik Thurin, Louise Carstam, Anja Smits, Sasha Gulati, Roger Henriksson, Øyvind Salvesen, Asgeir Store Jakola

**Affiliations:** 1 Department of Clinical Neuroscience, Institute of Neuroscience and Physiology, University of Gothenburg, Sahlgrenska Academy, Blå stråket 7, 3 tr, 413 45 Gothenburg, Sweden; 2 Department of Neurosurgery, Sahlgrenska University Hospital, Gothenburg, Sweden; 3 Department of Neurosurgery, St.Olavs University Hospital HF, Trondheim, Norway; 4 Department of Radiation Sciences and Oncology, University of Umea, Umeå, Sweden; 5 Department of Public Health and Nursing, Norwegian University of Science and Technology, Trondheim, Norway

**Correction to: BMC Cancer 21, 248 (2021)**

**https://doi.org/10.1186/s12885-021-07939-w**

Following publication of the original article [1], an error was discovered in table 3 “Logistic regression model for use of antidepressants, sedatives and anti-epileptics, respectively, at one year following index date, for patients with LGG.” In this table, the numbers presented for anti-epileptics are equal to the numbers for the sedatives. There has been a copy error and numbers need to be adjusted. The new results do not change the conclusions. The updated table 3 is given below and the changed values are displayed in bold typeface.

**Table:** 

Covariate	Univariable		Multivariable	
	OR (95 % CI)	***p***-value	OR (95 % CI)	***p***-value
**ANTIDEPRESSANTS**				
Index year (per year)	1.22 (1.07-1.39)	0.002**	1.31 (1.11-1.55)	0.002**
Female (vs. male)	2.99 (1.60-5.60)	0.001**	3.41 (1.71-6.80)	0.001**
Age (per year)	1.02 (1.00-1.05)	0.027*	1.02 (1.00-1.05)	0.11
Income (per 100.000 SEK)	0.96 (0.76-1.20)	0.69	1.07 (0.83-1.39)	0.60
Higher education (vs. lower education)	0.94 (0.78-1.15)	0.56	0.97 (0.78-1.21)	0.78
History of depression	7.61 (3.88-14.94)	<0.001***	6.12 (2.96-12.63)	<0.001***
Use of antidepressants preoperatively	14.15 (5.88-34.08),	<0.001***		
Elixhauser comorbidity index (0, 1, 2, >3)	1.33 (0.96-1.84)	0.09	1.09 (0.75-1.59)	0.65
Functional level (per WHO performance status category)	0.98 (0.92-1.03)	0.38	0.96 (0.88-1.06)	0.42
Tumour size (6)	1.08 (0.96-1-21)	0.22	0.92 (0.79-1.07)	0.30
**SEDATIVES**				
Index year (per year)	1.13 (0.99-1.29)	0.07	1.12 (0.97-1.30)	0.14
Female (vs. male)	1.23 (0.66-2.30)	0.51	1.13 (0.58-2.19)	0.73
Age (per year)	1.02 (1.00-1.04)	0.09	1.02 (0.99-1.04)	0.24
Income (per 100.000 SEK)	0.87 (0.67-1.13)	0.30	0.90 (0.62-1.22)	0.49
Higher education (vs. lower education)	0.81 (0.66-1.00)	0.05	0.90 (0.71-1.13)	0.35
History of anxiety and/or sleeping difficulties	2.58 (1.36-4.90)	0.004**	2.14 (1.09-4.20)	0.027*
use of sedatives preoperatively	3.05 (1.60-5.82),	<0.001***		
Elixhauser comorbidity index (0, 1, 2, >3)	1.37 (0.98-1.92)	0.07	1.17 (0.80-1.70)	0.41
Functional level (per WHO performance status category)	0.97 (0.91-1.04)	0.42	0.97 (0.89-1.05)	0.43
Tumour size (6)	1.09 (0.97-1.23)	0.17	1.00 (0.87-1.16)	0.98
**ANTI-EPILEPTICS**				
Index year (per year)	**1.00 (0.92-1.08)**	**1.00**	**1.01 (0.92-1.12)**	0.**83**
Female (vs. male)	1.**11** (0.**75-1.64)**	0.**60**	1.13 **(0.71-1.82)**	0.**60**
Age (per year)	1.0**0 (0.98**-1.0**1)**	0.**64**	1.0**0** (0.9**8**-1.0**2**)	0.**93**
Income (per 100.000 SEK)	**1.02** (0.**88**-1.1**8)**	0.**79**	0.9**4** (0.**79**-1.**1**2)	0.49
Higher education (vs. lower education)	**1.04** (0.**92**-1.**19**)	0.**53**	**1.04** (0.**87**-1.**2**3)	**0.69**
History of AED**-use and/or seizure/epilepsy preoperatively**	**12.16 (7.15-20.69)**	**<**0.001***	**12.98 (7.53-22.37)**	**<**0.001***
Elixhauser comorbidity index (0, 1, 2, >3)	**0.90** (0.**69**-1.**17**)	**0.42**	0.**93 (0.67-1.28)**	0.**64**
Functional level (per WHO performance status category)	**1.00** (0.9**9**-1.01)	0.**85**	**1.00** (0.**98**-1.0**1**)	**0.85**
Tumour size (6)	1.0**3** (0.9**4**-1.**12**)	0.**58**	1.00 (0.**90**-1.1**3**)	0.9**4**
** Biopsy vs resection**	**0.78 (0.49-1.25)**	**0.30**	**0.67 (0.38 –1.19)**	**0.17**

*p <0.05, **p < 0.01, ***p < 0.001
