# Supplementary material for: Psychotropic and anti-epileptic drug use, before and after surgery, among patients with low-grade glioma: a nationwide matched cohort study
Source: BMC Cancer. 2021 Mar 8;21:248. doi: 10.1186/s12885-021-07939-w (PMC7938599; doi:10.1186/s12885-021-07939-w)
Supplement: Supplementary file 1 — Additional file 1: Supplement Table 1. Characteristics of patients and controls. Supplement Table 2. changes in use of antidepressants for patients and controls. Supplement Table 3. Proportion (%) of users of antidepressants at one year after index date in relation to index year. Supplement Table 4. changes in use of sedatives for patients and controls. Supplement Table 5. changes in use of AEDs for patients and controls. [file 12885_2021_7939_MOESM1_ESM.docx]

**Supplement table 1. Characteristics of patients and controls.**

|  | **LGG (n= 485)** | **Controls (n=2412)** | **p-value** |
| --- | --- | --- | --- |
| Educational level |  |  | 0.79 |
| Basic to high-school | 285 (62.4) | 1482 (63.0) |  |
| Higher education | 172 (37.6) | 869 (36.9) |  |
| Missing, n | 28 | 61 |  |
| Disposable income index year, median (Q1-Q3) | 207929  (148101- 287155) | 223466  (146356-295256) | 0.29 |
| Number of Elixhauser comorbidities, n (%) |  |  | <0.001 |
| 0 | 362 (74.6) | 1995 (82.7) |  |
| 1 | 86 (17.7) | 262 (10.9) |  |
| 2 | 24 (4.9) | 85 (3.5) |  |
| ≥3 | 13 (2.7) | 70 (2.9) |  |
| Missing | 0 | 0 |  |

**Supplement Table 2: changes in use of antidepressants for patients and controls**

| **Group** | **Use of antidepressants**  **at index date** | **N (% of all patients)** | **Use at**  **1-year post-op.** | **N (% of subgroup)** |
| --- | --- | --- | --- | --- |
| **Patients**  **(485)** | Yes | 29 (6.0) | Yes | 14 (48.3) |
|  |  |  | No | 10 (34.5) |
|  |  |  | Dead | 5 (17.2) |
|  | No | 456 (94.0) | Yes | 37 (8.1) |
|  |  |  | No | 374 (82.0) |
|  |  |  | Dead | 45 (9.9) |
| **Controls**  **(2412)** | Yes | 146 (6.1) | Yes | 102 (69.9) |
|  |  |  | No | 41 (28.1) |
|  |  |  | Dead | 3 (2.1) |
|  | No | 2266 (93.9) | Yes | 57 (2.5) |
|  |  |  | No | 2199 (97.0) |
|  |  |  | Dead | 10 (0.4) |

**Supplement table 3. Proportion (%) of users of antidepressants at one year after index date in relation to index year.**

| **Index year** | **Controls** | **Patients** |  | |  | |
| --- | --- | --- | --- | --- | --- | --- |
| 2006 | 0.0 | 0.0 |  | <5 | |  |
| 2007 | 8.7 | 0.0 |  | <10 | |  |
| 2008 | 2.9 | 8.1 |  | <15 | |  |
| 2009 | 5.3 | 12.5 |  | <20 | |  |
| 2010 | 5.7 | 2.1 |  | ≥20 | |  |
| 2011 | 6.3 | 13.2 |  | |  | |
| 2012 | 6.5 | 15.9 |  | |  | |
| 2013 | 5.5 | 10.9 |  | |  | |
| 2014 | 8.7 | 16.7 |  | |  | |
| 2015 | 11.4 | 20.8 |  | |  | |

**Supplement table 4: changes in use of sedatives for patients and controls**

| **Group** | **Use of Sedatives**  **at index date** | **N (% of all patients)** | **Use at**  **1-year**  **post-op.** | **N (% of subgroup)** |
| --- | --- | --- | --- | --- |
| **Patients**  **(485)** | Yes | 108 (22.3) | Yes | 19 (17.6) |
|  |  |  | No  Dead | 78 (72.2)  11 (10.2) |
|  | No | 377 (77.7) | Yes | 25 (6.6) |
|  |  |  | No  Dead | 313 (83.0)  39 (10.3) |
| **Controls**  **(2412)** | Yes | 80 (3.3) | Yes | 43 (53.8) |
|  |  |  | No  Dead | 35 (43.8)  2 (2.5) |
|  | No | 2332 (96.7) | Yes | 41 (1.8) |
|  |  |  | No  Dead | 2280 (97.8)  11 (0.5) |

**Supplement table 5: changes in use of AEDs for patients and controls**

| **Group** | **Use of AEDs**  **at index date** | **N (% of all patients)** | **Use at**  **1-year post-op.** | **N (% of subgroup)** |
| --- | --- | --- | --- | --- |
| **Patients**  **(485)** | Yes | 285 (58.8) | Yes | 215 (75.4) |
|  |  |  | No  Dead | 50 (17.5)  20 (7.0) |
|  | No | 200 (41.2) | Yes | 53 (26.5) |
|  |  |  | No  Dead | 117 (58.5)  30 (15.0) |
| **Controls**  **(2412)** | Yes | 57 (2.4) | Yes | 46 (80.7) |
|  |  |  | No  Dead | 10 (17.5)  1 (1.8) |
|  | No | 2355 (97.6) | Yes | 11 (0.5) |
|  |  |  | No  Dead | 2332 (99.0)  12 (0.5) |
