# Supplementary figures and images for: Psychotropic and anti-epileptic drug use, before and after surgery, among patients with low-grade glioma: a nationwide matched cohort study
Source: BMC Cancer. 2021 Mar 8;21:248. doi: 10.1186/s12885-021-07939-w (PMC7938599; doi:10.1186/s12885-021-07939-w)

Supplement figure 1. Flow chart of patient selection

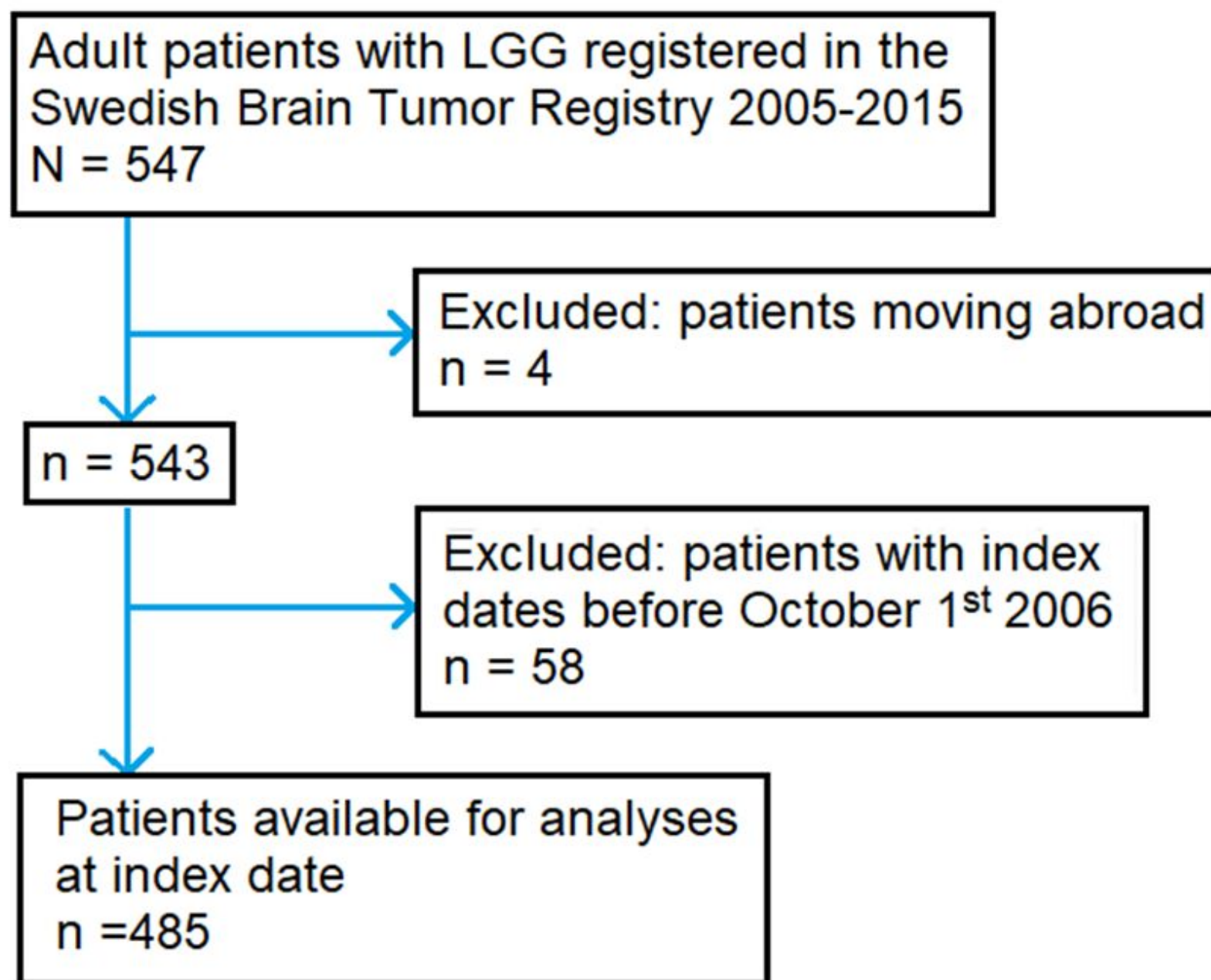

Supplement: Supplementary file 2 — Additional file 2: Supplement Fig. 1. Flow chart of patient selection. [file 12885_2021_7939_MOESM2_ESM.pdf]
